# Measuring Neighborhood Order and Disorder: a Rapid Literature Review

**DOI:** 10.1007/s40572-019-00259-z

**Published:** 2019-11-26

**Authors:** Steeve Ndjila, Gina S. Lovasi, Dustin Fry, Amélia A. Friche

**Affiliations:** 1 Urban Health Collaborative, Dornsife School of Public Health, Drexel University, 3600 Market St, 7th Floor, Philadelphia, PA 19104 USA; 2 Belo Horizonte Observatory for Urban Health, School of Medicine, Federal University of Minas Gerais, Belo Horizonte, Brazil

**Keywords:** Neighborhood disorder, Neighborhood environments, Street observations, Virtual audits, Physical disorder

## Abstract

**Purpose of Review:**

Neighborhood disorder has received attention as a determinant of health in urban contexts, through pathways that include psychosocial stress, perceived safety, and physical activity. This review provides a summary of data collection methods, descriptive terms, and specific items employed to assess neighborhood disorder/order.

**Recent Findings:**

The proliferation of methods and terminology employed in measuring neighborhood disorder (or neighborhood order) noted over the past two decades has made related studies increasingly difficult to compare. Following a search of peer-reviewed articles published from January 1998 to May 2018, this rapid literature review identified 18 studies that described neighborhood environments, yielding 23 broad terms related to neighborhood disorder/order, and a total of 74 distinct measurable items.

**Summary:**

A majority of neighborhood disorder/order measurements were assessed using primary data collection, often relying on resident self-report or investigatory observations conducted in person or using stored images for virtual audits. Items were balanced across signs of order or disorder, and further classification was proposed based on whether items were physically observable and relatively stable over time.

## Introduction

Neighborhood conditions are increasingly recognized as having an important impact on the health of neighborhood residents beyond what can be explained by individual-level characteristics alone [1]. Neighborhood characteristics have therefore gained attention in health-related research for several psychosocial and behavioral pathways over the past two decades [2–5]. Adding to this momentum, *Exposure Science in the 21*^*st*^*Century: A Vision and a Strategy* report released in 2012 by the National Research Council (NRC) pointed out a need for more comprehensive exposure data collection procedures that include environmental and community characteristics in addition to individual-level exposures [6].

As the body of literature on neighborhood characteristics has grown, so has the list of terms used to describe these characteristics, and several distinct classification schemes have been proposed [2–5, 7, 8, 9•, 10–12]. Given this proliferation and diversification of neighborhood-assessment tools and terminology, distinctions and relationships between concepts can be complex to navigate, making related research challenging to identify and interpret. Consolidation and clear delineation of concepts are made even more important by the emergence of multinational collaborations such as the Salud Urbana en América Latina (Urban Health in Latin America, SALURBAL) project [13••] which includes spatial and temporal comparisons relevant to understanding how urban environments affect population health [14•, 15•]. Quistberg, Roux [16••] further emphasizes the necessity for ensuring comparability of measures across various secondary data from distinct urban settings (cities and sub-cities). However, secondary data are not uniformly available for all aspects of health-relevant urban environmental variation, particularly for indirectly measured concepts such as neighborhood disorder.

Neighborhood disorder/order has emerged as a particularly prominent term that is cited in a large collection of health-related research [3, 7, 9•, 10, 17]. For example, Latkin, Curry [3] report direct associations between neighborhood disorder indicators such as vandalism, littering and/or loitering, and high-risk substance use and sexual behavior patterns. Although the concept of neighborhood disorder is used extensively, it is not always defined explicitly. Available literature shows a gradual evolution of the framing of physical disorder as a potential signal of social context and determinant of health. One of the earliest views of the term neighborhood disorder defines it as a pattern of divergence away from conventionally accepted norms or standards within a community [18]. This may be manifested as the perceptible decay of the urban scenery or the proliferation of uncivil social behavior and resultant physical signs such as broken windows or an accumulation of litter [19–21]. Ross and Jang [22] built on this early view and introduced a second perspective that highlights the presence of measurable neighborhood processes or items such as vandalized or abandoned property (including both vacant lots, buildings, and vehicles) as indicators of neighborhood disorder. This work brought to prominence the idea that neighborhood disorder is not always criminal in nature but is inclusive of a range of criminalized and non-criminal factors that indicate substandard neighborhood maintenance or affinity such as graffiti, buildings in states of disrepair, and loitering. Today, a third and more prominent view of neighborhood disorder focuses more on perception of the neighborhood by residents as a stressor, incorporating a more subjective lens. Under this definition, neighborhood disorder is described as a generally perceived lack of order and social control within a community [23]. Neighborhood residents and/or investigators see visible cues and decide whether to interpret them as indicators of neighborhood disorder based on their preconceptions. This allows for awareness of how subjectivity can influence ratings, as the same neighborhood feature could be viewed as indicating disorder by one viewer but not by another. Even when residents or researchers would agree that an item indicates disorder, there may be disagreement about the degree to which disorder is perceived (slight to severe). For pathways involving resident stress-related or behavioral responses to the environment, attention to how residents (vs researchers) perceive the environment may be particularly crucial.

In this review, we aim to provide an orientation to some common terms used in describing neighborhoods, including broader terms related to the disorder/order spectrum and the specific items measured to characterize these terms. This will help contextualize current findings and guide the description and consolidation of measurement strategies which to date have been highly variable.

## Study Design

A rapid review of the literature on neighborhood disorder and health was conducted to identify common terminology and to provide guidance on measurement options relevant to future data collection for neighborhood-scale investigations globally.

### Identification and Inclusion of Papers

To begin the search, the terms “neighborhood disorder” and “physical disorder” were in turn entered in a search box that was restricted to article titles only on the National Center for Biotechnology Information (NCBI) web database, PubMed. Peer-reviewed English language articles published from the year 1998 through May 2018 (20 years span) were then selected through a snowball approach [24] starting with a recently published original research article by Robinette, Charles [25••] published in *Social Science and Medicine* in 2018. Informed by this search, cited articles with similar and related terms were also identified.

**Fig. 1:**
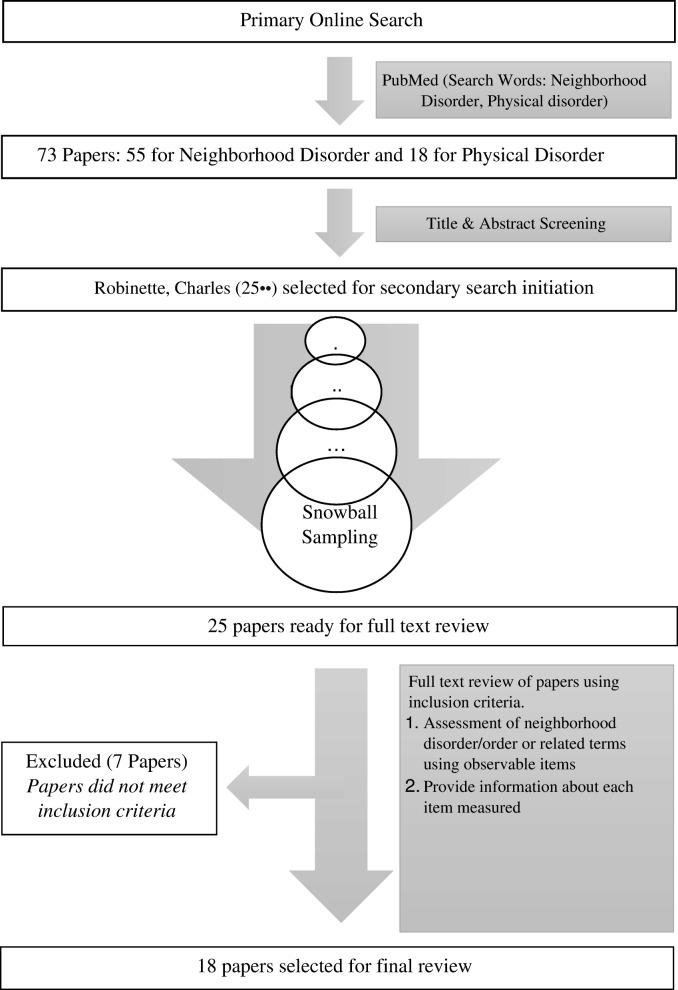
Identification and inclusion of papers in this rapid review

Inclusion criteria were (1) assessment of neighborhood disorder/order and related terms using measurable items (or descriptions) via primary data (in-person, virtual, and/or self-report) and/or secondary data sources and (2) complete information about assessed neighborhood characteristics (reporting all street level items used to assess each neighborhood characteristic) (Fig. 1).

### Characterization of Data Collection Methods and Included Items

Information abstracted from included articles allowed categorization of data collection methods and identification of unique terms and items (Table 1). We distinguished methods to characterize the neighborhood environment as primary (collected by the investigators for research purposes) or secondary data (available from prior research or surveillance efforts, commonly including publicly available data). Studies that used primary data were further categorized with attention to the groupings relevant to whether residents or investigators were engaged in measurement and whether any systematic observation was in-person or virtual. In-person data collection included only studies that trained people to conduct data collection via systematic in-person observations in the neighborhoods of interest. Examples include Kelly, Schootman [11], Wei, Hipwell [26], and Douglas, Briones [9•]. Another such study beyond the scope of our search (screened out prior to full inclusion criteria assessment due to our restriction to English language publications) is Costa, Mingoti [27]. Virtual audit data collection included studies that made use of stored imagery such as Google Street View images. Examples include Marco, Gracia [12], Mooney, Bader [28], and Sampson and Raudenbush [21]. Self-report included surveys or interviews with residents reflecting on the characteristics of their neighborhood, an area for which the boundaries were often not explicitly specified. An example of a study that used this data collection procedure is Oropesa [29]. Other similar studies beyond the scope of our search (due to our restriction to English language publications) include de Almeida Célio, de Lima Friche [30] and Andrade, Peixoto [31].

For each article included in this review, all reported items used in measuring neighborhood characteristics were extracted and initially categorized using terms drawn from the articles themselves. The same specific item could be grouped under multiple broader terms by different articles. Some very similar items were different only based on measuring presence vs absence, and thus could be considered as the inverse (or reverse coding) of each other. For example, Zandieh, Martinez [32] measured litter, placing emphasis on the absence of litter to signal neighborhood order, while Kelly, Schootman [11] also measured litter but focused on presence of litter to indicate neighborhood disorder. To simplify our representation of the items, descriptive terms such as good, bad, high level of, or presence/absence were omitted to distill a shorter list of measurable items. Hence, for both studies named above, the item extracted was “litter/trash/rubbish.” Articles from which the items were drawn were also noted such that each item was associated with an original source reference.

Following this initial extraction of items, the team of authors developed through consensus a stratification system based on three ways to divide the items: (1) order/disorder, whereby each item was determined as indicating either order or disorder; (2) physical/social, whereby each item was assessed for whether it would be apparent through observing the physical environment or through social dynamics; and (3) temporary/stable, whereby each item was assessed for likely short-term variation (hours, days, or weeks) or relative stability (though still subject to longer-term transitions, stable items were thought to be less sensitive to the exact timing of observation). This scheme to stratify items was devised with attention to both capturing a range of positive and negative aspects of urban areas, as well as to show how the nature of items might restrict our options for measurement. For example, physical factors such as litter and graffiti may be more suitable to systematic observation, whereas social dynamics such as trust in neighbors and community unity may not be as readily observable by investigators using virtual or even in-person audits. Although all aspects of neighborhood disorder in general have social causes and psychosocial consequences, not all are detectible from the visible features of the environment. Finally, our classification of items as relatively temporary versus stable is relevant to reliability in capturing a state of the environment such as noise or litter which can vary throughout the day or week. Further refinement to our assessment of which items are stable may be particularly well captured through carefully timed, repeated in-person audits. Where short-term fluctuations are relatively large, the timing of virtual audits that rely on available imagery may be an important limitation. Likewise, self-reported neighborhood characteristics that generally rely on observations over an unspecified period may mask important variation over time. Stable items such as deteriorated buildings may be more reliably observable across a range of data collection techniques, while still being amenable to deliberate community investment efforts such as urban redevelopment (Table 1).

**Table 1:** Characteristics of articles reviewed

Study	Type of data collected	Primary data collection protocol	Party assessing disorder/order
Bowling, Barber, Morris, and Ebrahim (2006)	Primary data	Self-report (interviews)	Participants
Cunradi (2009)	Primary data	Self-report (interviews)	Participants
Latkin, Curry, Hua, and Davey (2007)	Primary data	Self-report (interviews)	Participants
Latkin et al. (2017)	Primary data	Self-report (interviews)	Participants
Litt et al. (2011)	Primary data	Self-report (interviews)	Participants
Miles (2008)	Primary data	Self-report (interviews) and in-person	Participants and investigators
Oropesa (2012)	Primary data	Self-report (interviews)	Participants
Robinette, Charles, and Gruenewald (2018)	Primary data	Self-report (interviews)	Participants
Ross and Mirowsky (2001)	Both primary and secondary data	Self-report (interviews)	Participants
Zandieh, Martinez, Flacke, Jones, and Van Maarseveen (2016)	Primary data	Self-report (interviews)	Participants
Douglas et al. (2018)	Primary data	In-person	Investigators
Kelly, Schootman, Baker, Barnidge, and Lemes (2007)	Primary data	In-person	Investigators
Wei, Hipwell, Pardini, Beyers, and Loeber (2005)	Both primary and secondary data	In-person	Investigators
Marco, Gracia, Martín-Fernández, and López-Quílez (2017)	Primary data	Virtual	Investigators
Mooney et al. (2014)	Primary data:	Virtual	Investigators
Sampson and Raudenbush (1999)	Primary data	Virtual	Investigators
Cerdá et al. (2009)	Secondary data	N/A	N/A
Mason et al. (2017)	Secondary data	N/A	N/A

Primary data refers to data collected by the investigators for research purposes

Secondary data refers to data available from prior research or surveillance efforts, commonly including publicly available data

## Current Findings

The initial title search yielded 73 results in total: 55 for the term “neighborhood disorder” and 18 for the term “physical disorder.” After screening titles to determine which papers assessed neighborhood disorder/order and using a snowball sampling methodology, 25 papers were selected for review. After full text review of these selected papers, 18 met our inclusion criteria.

The review yielded 23 distinct terms (including neighborhood disorder/order themselves) used to describe neighborhood environments with a total of 74 specific items measured to assess them (Table 2).

**Table 2:** List of street-level items categorized by terms

Term	Number of studies using this term	Street-level item measured
Physical disorder/order [8, 11, 12, 21, 25••, 26, 28, 33, 34]	9	Abandoned vehicles Auditory annoyance (noise) Bar-windowed buildings Broken glass/windows Cigarette butts Cleanliness Deteriorated buildings Empty bottles (beer or liquor) Graffiti (with or without political message or protest) and graffiti painted over House maintenance Litter/ trash/ rubbish Needles/ syringes Sex Paraphernalia Vacant/abandoned buildings (homes and others) Vacant/abandoned buildings (homes and others) Vacant/abandoned or undeveloped land Vandalism Vandalized or run-down buildings Vegetation (artificial and man-made) Cleanliness
Social disorder/order [21, 25••, 29, 33]	4	Crime (assaults, robbery, muggings…) Drug use and/or trafficking Gangs Respect for rules, laws, and authority Perceived nighttime street safety Loitering Alcohol use Street fights (and disputes) Prostitution interpersonal relationships Willingness to help neighbors Perceived neighborhood safety
Neighborhood disorder/order [5, 9, 17, 35]	4	Alcohol use Auditory annoyance (noise) Broken glass/windows Crime (assaults, robbery, muggings…) Dog refuse Drug use and/or trafficking Graffiti (with or without political message or protest) and graffiti painted over Litter/ trash/ rubbish Loitering Owner-occupied housing Poverty (household and individual) Sex Paraphernalia Single-parent households Street fights (and disputes) Vacant/abandoned buildings (homes and others) Vandalism Vegetation (artificial and man-made)
Neighborhood aesthetics [4, 32]	2	Attractive sites (natural and man-made) Litter/ trash/ rubbish Shade Vegetation (artificial and man-made) Well-maintained front gardens
Neighborhood safety [29, 32]	2	Crime (assaults, robbery, muggings…) Pedestrian interaction Pedestrian visibility Perceived daytime street safety Perceived nighttime street safety Street lighting
Neighborhood air quality [32]	1	Exhaust fumes
Neighborhood amenities [32]	1	Public benches Public toilets Shelters
Neighborhood attachment [4]	1	Emotional attachment to neighborhood facilities Sense of belonging to neighborhood
Neighborhood characteristics [26]	1	Minority concentration Poverty (household and individual) Vacant/abandoned buildings (homes and others)
Neighborhood cohesion [25••]	1	Interpersonal solidarity Sense of belonging to neighborhood
Neighborhood disadvantage [33]	1	Adults 25+ with college degrees Mother-only households Owner-occupied housing Poverty (household and individual)
Neighborhood Interaction (social cohesiveness or neighborhood cohesiveness) [29]	1	Community unity Interpersonal professional discussions interpersonal relationships Interpersonal social visits Trust in neighbors Willingness to help neighbors
Neighborhood political engagement [2]	1	Participation in elections
Neighborhood problems [2]	1	Air quality Auditory annoyance (noise) Crime (assaults, robbery, muggings…) Graffiti (with or without political message or protest) and graffiti painted over Litter/ trash/ rubbish Speed/volume of traffic (including nearby streets)
Neighborhood quietness [32]	1	Auditory annoyance (noise)
Neighborhood sidewalks [11]	1	Sidewalk walkability Sidewalks
Neighborhood social involvement [4]	1	Advocacy for neighborhood issues Participation in local activities Participation in neighborhood meetings
Neighborhood traffic condition [32]	1	Crosswalks and pedestrian signaling Perceived safety of crosswalks Respect of driving rules Speed/volume of traffic (including nearby streets)
Neighborliness [2]	1	interpersonal relationships Perceived nighttime street safety Trust in neighbors
Perceived neighborhood disorder/order [3]	1	Crime (assaults, robbery, muggings…) Drug use and/or trafficking Litter/ trash/ rubbish Loitering Vacant/abandoned buildings (homes and others) Vandalism
Perceived neighborhood environment [2]	1	Attractive sites (natural and man-made) Commercial facilities (shops) Facilities for people aged 65+ Leisure/social facilities Local health services Rubbish collection Transport
Perceived neighborhood safety [34]	1	Perceived nighttime street safety
Physical decay [12]	1	Deteriorated recreation places Deteriorated residential units Vacant/abandoned buildings (homes and others) Vandalized or run-down buildings

Stratifying the 74 items (disorder/order, physical/social, or temporary/stable) yielded the following results: 43 items described *order* and related concepts, while the remaining 31 items described *disorder* and related concepts; 36 items fell under the category *physical*, while the remaining 38 items fell under the category *social*; 31 items fell under the category *temporary*, while the remaining 43 items fell under the category *stable* (Table 3). We note that there may be efforts needed to avoid conflation of neighborhood social disorder with commonly measured social determinants of health based on population characteristics.

**Table 3:** List of street-level items categorized by descriptive category

		Physical	Social
Order (absence of these items indicates disorders)	Temporary	• Cleanliness • Shade • Shelters • Sidewalk walkability • Vegetation (artificial and man-made) • Well-maintained front gardens	• Community unity • Interpersonal professional discussions • Interpersonal social visits • Participation in neighborhood meetings • Pedestrian interaction • Pedestrian visibility • Perceived daytime street safety • Perceived nighttime street safety • Respect of driving rules • Willingness to help neighbors
	Stable	• Air quality • Attractive sites (natural and Man-made) • Commercial facilities (shops) • Crosswalks and pedestrian signaling • House maintenance • Leisure/social facilities • Local health services • Owner-occupied housing • Public benches • Public toilets • Sidewalks • Street lighting • Transport	• Adults 25+ with college degrees • Advocacy for neighborhood issues • Emotional attachment to neighborhood facilities • Facilities for people aged 65+ • interpersonal relationships • Interpersonal solidarity • Participation in elections • Participation in local activities • Perceived neighborhood safety • Perceived safety of crosswalks • Respect for rules, laws, and authority • Rubbish collection • Sense of belonging to neighborhood • Trust in neighbors
Disorder (presence of these items indicates disorder)	Temporary	• Abandoned vehicles • Broken glass/windows • Cigarette butts • Dog refuse • Empty bottles (beer or liquor) • Litter/ trash/ rubbish • Needles/ syringes • Sex Paraphernalia	• Alcohol use • Auditory annoyance (noise) • Drug use and/or trafficking • Gangs • Loitering • Speed/volume of traffic (including nearby streets) • Street fights (and disputes)
	Stable	• Deteriorated buildings • Deteriorated recreation places • Deteriorated residential units • Exhaust fumes • Graffiti (with or without political message or protest) and graffiti painted over • Vacant/abandoned buildings (homes and others) • Vacant/abandoned or undeveloped land • Vandalism • Vandalized or run-down buildings	• Bar-windowed buildings • Crime (assaults, robbery, muggings…) • Minority concentration • Mother-only households • Poverty (household and individual) • Prostitution • Single-parent households

Note: Designation as temporary or stable is provisionally assigned but empirically testable and should be reevaluated in future work

## Discussion

During the categorization of the 23 neighborhood disorder/order related terms identified (such as neighborhood aesthetics, physical decay, and neighborhood cohesion), we noted that different data collection methods were varyingly suited to certain groups of street-level items. For example, relatively stable items and those capturing aspects of the physical environment are amenable to data collection using virtual audits, whereas social disorder and related social environment characteristics are more amenable to data collection using self-report or ecometric (a combination of socio-economic and environmental) measures. Across data collection approaches, we note the potential to characterize a spectrum from items signaling order/care to those signaling disorder/deterioration.

### Implications

The broad range of terminology obtained from this brief review is important to understand given the rapid growth of interest in measuring and describing neighborhood characteristics. A majority of neighborhood disorder/order measurements were assessed using primary data collection, often relying on resident self-report or investigatory observations conducted in person or using stored images for virtual audits. Items were balanced across signs of order or disorder, and further classification was proposed based on whether items were physically observable and relatively stable over time.

Research focused more on items posited to be stable rather than temporary. Empirical observation can be used to refine our classification of which items are observed to exhibit stability across months, years, and even decades.

Neighborhood disorder, often broken down into two distinct constructs (physical disorder and social disorder) [18, 36], is closely related to other terms (some of which have been identified in this review) that have emerged in recent literature, including neighborhood aesthetics, physical decay, and social cohesion [4, 10, 12, 25••]. Therefore, distinctions and relationships between these concepts can be ambiguous, making related research challenging to assemble and interpret. A more standard application of terminology is needed to reduce the ambiguity often associated with the use of these concepts in research.

Even though our snowball sampling was initiated with the terms physical and neighborhood disorder, we did not include only studies using these specific two terms. The methodology employed entailed actively searching for and reviewing papers that used synonymous or related terms to describe neighborhood environments. We are, however, aware that this strategy made it more likely than not to capture articles that employed these two specific terms, so the proportions of studies under each term in Table 2 should not be taken as representative of the broader literature.

Planned neighborhood observations may benefit from considering whether aspects such as the temporal permanence (temporary/stable) and the physical observability (physical/social) of the specific items is well matched to the measurement strategy, and considering strategies to improve the accuracy and precision of these measurements. As physical disorder/order assessment is extended to new settings, individual items may need to be assessed for differential item functioning and for alignment with what residents understand as representing physical disorder/order. For example, vegetation may be an indicator of disorder in rural settings but an indicator of order in urban settings. Also, graffiti could in some instances be part of urban renovation in informal settlements and may be considered as art potentially indicating order. Hence, certain items may need to be adapted prior to measurement depending on the setting. In addition, many items relevant to physical disorder/order incorporate subjective evaluation such as distinguishing between graffiti and a mural based on aesthetic value and inferred purpose, further rendering the systematization of protocols more challenging.

### Strengths and Limitations

The current rapid review provides an orientation to the data collection methods, terms, and items commonly used in health-relevant research on neighborhood disorder/order. However, our focus on title searching followed by a snowball approach to expanding the pool of included articles was not comprehensive, and there may be additional available terminology and measurable items that warrant consideration for future work describing neighborhoods. Although the included articles suggest a wide range of terms and items have been used, this review may have omitted literature with relevance to the subject matter and thus underestimated the heterogeneity of terms and items used.

## Conclusions

Understanding the influence of neighborhood disorder/order on population health is challenging due to the diversity of terms and items used. Clear definitions and consolidation of terminology in the neighborhood disorder/order literature would facilitate comparisons and synthesis across related studies. Efforts toward standardization of research and terminology on the neighborhood disorder/order concept may benefit from consolidating measurement items within our proposed strata, as well as refinement of how items are classified and empirical investigation of how items are most reliably measured. Where specific settings require the inclusion of more novel or tailored items, these could be used alongside a common set of items to ease comparisons across settings and clarify the added value of setting-specific additions.
